# Diet Outcomes from a Randomized Controlled Trial Assessing a Parenting Intervention Simultaneously Targeting Healthy Eating and Substance Use Prevention among Hispanic Middle-School Adolescents

**DOI:** 10.3390/nu15173790

**Published:** 2023-08-30

**Authors:** Sonia Vega-López, Stephanie Ayers, Anaid Gonzalvez, Ana Paola Campos, Flavio F. Marsiglia, Meg Bruening, Lela Rankin, Beatriz Vega Luna, Elizabeth Biggs, Alex Perilla

**Affiliations:** 1 College of Health Solutions, Arizona State University, Phoenix, AZ 85004, USA; 2 Southwest Interdisciplinary Research Center, Arizona State University, Phoenix, AZ 85004, USA; stephanie.l.ayers@asu.edu (S.A.); anaid.gonzalvez@asu.edu (A.G.); paola.campos@asu.edu (A.P.C.); lela.rankin@asu.edu (L.R.); irmavegadel@asu.edu (B.V.L.); egonza69@asu.edu (E.B.); 3 Global Center for Applied Health Research, Arizona State University, Phoenix, AZ 85004, USA; marsiglia@asu.edu; 4 Department of Nutritional Sciences, College of Health and Human Development, The Pennsylvania State University, University Park, PA 16802, USA; mmb203@psu.edu; 5 WeGrad (Formerly American Dream Academy), Arizona State University, Phoenix, AZ 85004, USA; aperilla@asu.edu

**Keywords:** adolescents, diet, families, Hispanic, parenting intervention

## Abstract

Parents play a significant role in adolescent health behaviors; however, few nutrition interventions for Hispanic adolescents involve parents. This study assessed the effects of a 10-week parenting intervention simultaneously targeting nutrition and substance use prevention. Hispanic parent/6th–8th-grade adolescent dyads (n = 239) were randomized to Families Preparing the New Generation Plus (FPNG+; nutrition/substance use prevention), FPNG (substance use prevention only), or Realizing the American Dream (RAD; academic success control). Surveys assessed diet, alcohol use, substance use intentions, and substance use norms at baseline (T1), immediately post-intervention (T2), and at 16 weeks post-intervention (T3). Latent change modeling assessed diet changes; adolescent substance use outcomes were assessed using effect sizes. Among adolescents, those in FPNG+ increased fruit (+0.32 cup equivalents, *p* = 0.022) and fiber intake (+1.06 g, *p* = 0.048) and did not change added sugars intake at T2; those in FPNG and RAD reduced their intake of fruit and fiber (*p* < 0.05 for both). FPNG+ parents marginally increased fruit/vegetable intake (+0.17 cup equivalents, *p* = 0.054) and increased whole grains intake (+0.25-ounce equivalents, *p* < 0.05), in contrast to the reduction among RAD and FPNG parents (*p* < 0.05). Reductions in added sugar intake at T2 were greater among FPNG and FPNG+ parents relative to RAD parents (*p* < 0.05). FPNG+ and FPNG had comparable substance use outcomes (i.e., both had lower alcohol use and intentions to use substances relative to RAD). Engaging parents in a nutrition and substance use prevention parenting intervention yielded positive changes in dietary intake and maintained substance use prevention outcomes among their adolescent children.

## 1. Introduction

Individuals of Hispanic descent, now identified as the largest ethnic minority group in the United States (US) [1], have an increased risk for obesity and associated chronic conditions (e.g., diabetes, cardiovascular disease, and cancer) relative to their white counterparts [2,3]. This increased risk is in part associated with lifestyle-related factors, such as following poor dietary habits, lack of physical activity, and use of alcohol [4,5], leading to obesity and its comorbidities. In addition to lifestyle-related factors, Hispanic families tend to have lower educational level and socioeconomic status than non-Hispanic populations, and social determinants of health also impact nutrition-related outcomes [6]. Of particular concern is the risk for chronic diseases resulting from the increased prevalence of obesity among Hispanic children and adolescents, which in 2017–2018 was 1.6 times higher than that of their non-Hispanic white counterparts [7,8]. Therefore, there is a great need for interventions to help reverse this trend.

Low diet quality is an important factor contributing to chronic disease risk [9,10]. Adherence to dietary recommendations, often assessed using the Healthy Eating Index (HEI) [11], tends to be low across multiple population subgroups, including Hispanics [12,13,14]. HEI tends to drop to its lowest scores during adolescence among all racial/ethnic groups [14]. Whereas Hispanic adolescents have been reported to have higher HEI-2015 values than adolescents from other racial/ethnic groups, their scores (<50 out of 100 possible points) are indicative of a need for significant diet improvement [15,16]. For example, data from the 2007–2014 National Health and Nutrition Examination Survey (NHANES) suggest that although Hispanic adolescents consume greater amounts of fruit and vegetables than their non-Hispanic counterparts, their HEI-2015 adequacy scores for fruits and vegetables are at about 50% of the recommended intake [15]. Similarly, NHANES 2011-2014 data indicate that Hispanic children and adolescents (2–19 years) have a lower caloric intake from sugar-sweetened beverages than their non-Hispanic white and non-Hispanic Black counterparts, but these beverages still contribute about 7.3% and 6.8% of the daily energy intake for boys and girls, respectively [17]. Furthermore, it has been reported that Hispanic adolescents have a high intake of snacks (>20% of their daily energy intake) [18] and ultra-processed foods (~64% of their daily energy intake) [19], both of which have been associated with obesity among Mexican-origin children [20,21]. All these dietary traits suggest an urgent need for strategies to improve diet and related behaviors in Hispanic adolescents.

Diet-related behaviors, like many other health behaviors, are shared among different family members [22,23]. Parents largely shape adolescent dietary practices given their role as providers of foods and other resources for the family and their ability to role model diet-related behaviors [24,25]. Despite the role of family-level factors on diet-related behaviors and the potential benefit of involving multiple family members in behavior change efforts [26,27], diet improvement and obesity prevention interventions for Hispanic adolescents have traditionally only involved the adolescents [28,29]. Effective parenting practices are associated with positive health behaviors, such as healthy eating [30], and reduced risk for negative health behaviors, such as substance use [31,32]. In fact, Families Preparing the New Generation (FPNG), a substance use prevention parenting intervention, has been shown to improve family functioning, strengthen parent–child communication, and delay substance use initiation among Hispanic families [33,34,35,36]. However, the impact of a parenting intervention on diet-related behaviors has not been tested. 

The purpose of this study was to assess if Families Preparing the New Generation Plus (FPNG+), a parenting intervention designed to target adolescent healthy eating and substance use prevention simultaneously, can be efficacious with two different outcomes: healthy eating (i.e., estimated intake of fruit, vegetables/legumes, whole grains, fiber, dairy, and added sugars overall and from beverages) among participating parents and their adolescent children, as well as adolescent-reported substance use-related outcomes. The FPNG+ intervention is guided by the ecodevelopment theory [37,38], which organizes the multiple influences on adolescents from microsystems like the family, a context where the adolescent participates directly to macrosystems, which include broader influences such as culture and acculturation [39]. The working hypothesis for the present study is that parents and adolescents receiving a culturally congruent intervention targeting healthy eating and substance use prevention will strengthen their healthy eating significantly more than those in the comparison group and adolescents will report similar desired results on drug use outcomes than the original substance use only intervention.

## 2. Materials and Methods

### 2.1. Study Design

Dyads of one adult parent and one 6th–8th grade (11–14 years old) adolescent child from the same household were enrolled in a 3-arm parallel design cluster randomized controlled trial aimed at testing the efficacy of Families Preparing the New Generation Plus (FPNG+), a parenting intervention designed to target adolescent healthy eating and substance use prevention simultaneously. Participant dyads were recruited between January 2019 and February 2020 from local public middle schools and randomized at the school level to the FPNG+ intervention, the original FPNG intervention targeting substance use prevention only, or the Realizing the American Dream (RAD) intervention, a control intervention focusing on academic success that did not include content related to healthy eating or substance use prevention strategies. Data were collected from parents and adolescents via survey at baseline (T1), at the end of the 10-week interventions (T2), and at 16 weeks post-intervention (±2 weeks; T3). A more detailed description of the study design has been published elsewhere [40]. All study materials and procedures were approved by the Institutional Review Board at Arizona State University (STUDY00006797). The study is registered at ClinicalTrials.org (Identifier # NCT03517111).

### 2.2. Schools and Participants

Schools were eligible to participate if they met the following criteria: (1) public school offering 6th, 7th, and 8th grades with at least 65 students per grade; (2) located in Maricopa County, AZ; (3) having a Hispanic student population of at least 60%; and (4) receiving Title I funds from the federal government to assist in meeting students’ educational needs. A total of 84 schools met the eligibility criteria; of those, the first 36 schools with decreasing numbers of student enrollment were then clustered by geographical location. Within each of the 12 geographic clusters (3 schools per cluster), each school was assigned a random number generated by Excel. Performed by the study methodologist, it was determined a priori that within each cluster, the school with the lowest random number would be assigned to the FPNG+ intervention, next lowest to FPNG, and the third lowest to RAD. This sequence was repeated for all 12 clusters and 36 schools. The allocation sequence was concealed from all other study team members until interventions were assigned. Eligible schools were contacted by a community partner to ask if they were willing to offer the assigned program to their students’ parents at their facilities. Schools were no longer contacted once the team had enrolled 6 schools for a given study arm, or until study implementation ended due to the COVID-19 pandemic. A total of 16 local middle schools were part of the study: 6 were randomized to the FPNG+ intervention, 5 to the original FPNG intervention, and 5 to the RAD control intervention. Of the 16 schools, 3 schools joined the study for the first time in the spring of 2020 and are not included in the present analysis because the team was unable to deliver the full intervention and collect T2 or T3 data after February 2020 due to the COVID-19 pandemic. Therefore, the current analysis includes data from 13 schools (n = 5 for FPNG+; n = 3 for FPNG Original; n = 5 for RAD) and only includes data from the participants in the cohorts that received the full intervention.

**Figure 1:**
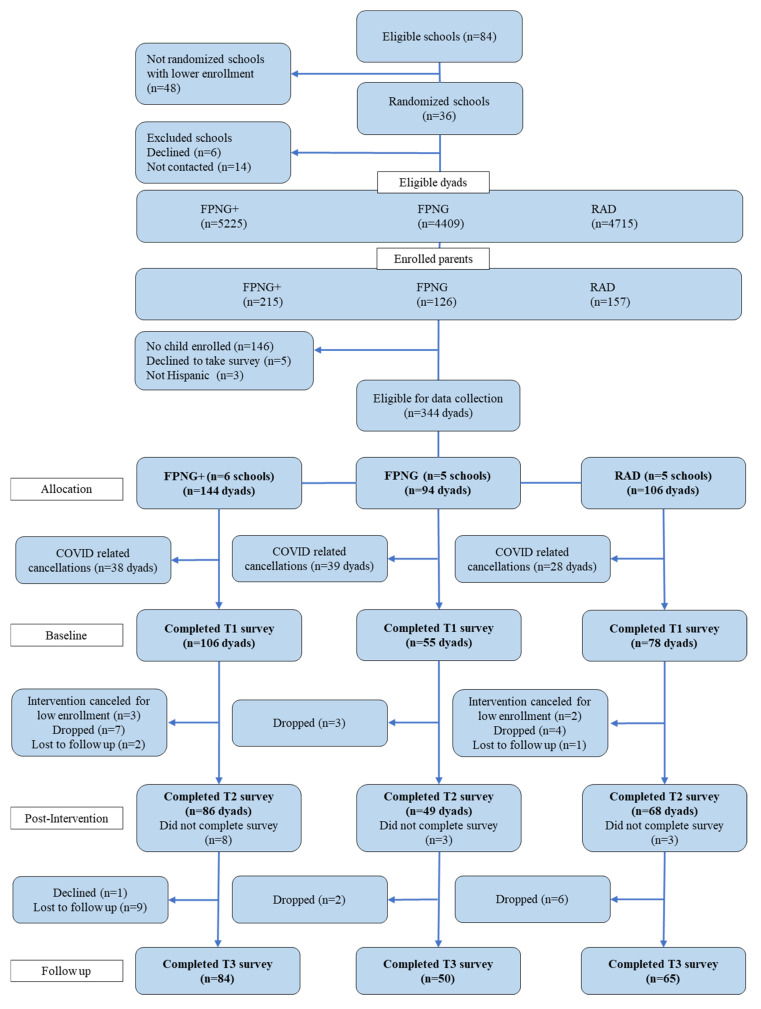
CONSORT diagram depicting the flow of the participants in the study.

Participating dyads were eligible if parents were 18 years or older, their adolescent child was enrolled in 6th, 7th, or 8th grade of a participating school at the time of recruitment, and were, self-reported, of Hispanic/Latinx descent. Participating adults provided written informed consent and parental permission for their adolescent child to take part in the study; participating adolescents provided written assent prior to participation. Adolescent participation was not required for parents to enroll in the study. Based on school enrollment data during the open recruitment phases of the study, there were a total of 14,349 students/parent dyads in participating schools. This yielded 496 eligible parents who were enrolled in the study, of which 344 provided parental consent and adolescent assent to participate. Of those, 105 dyads were excluded from this analysis due to COVID-19-related cancellations of the intervention programs, leaving data from 239 dyads for the present analysis. Complete follow-up data is available for 205 dyads at T2 and for 203 dyads at T3. The CONSORT flow diagram (Figure 1) shows the school and the participant enrollment per intervention group.

### 2.3. Intervention Programs

A detailed description of the three manualized interventions is available elsewhere [40]. Briefly, the three 10-week group-based interventions were delivered in Spanish to parents. All interventions were delivered by trained community facilitators at the school where the adolescents attended. The original FPNG program is an efficacious parenting intervention designed to prevent Hispanic adolescent substance use by providing parents with strategies for effective parent–child communication and improved family functioning [33,35]. The FPNG+ program included the same topics as its original counterpart, and additional content focuses on healthy eating and parenting strategies to promote healthy eating behaviors among all members of the family. The control RAD program focused on helping participants’ children achieve academic success through parental involvement in their children’s education and planning for an academic path towards enrolling in college.

### 2.4. Measures

Data were collected at all time points from parents and adolescents via a self-administered Qualtrics-based (Qualtrics, Provo, UT, USA) electronic survey accessed through Lenovo Tab 10 [TB-X103F] tablets (Lenovo Group Limited, Morrisville, NC, USA), which were made available to participants at the time of data collection. Surveys were available in Spanish and English, so participants were able to complete them in their language of preference. Bilingual study personnel were available at all data collection points to assist with tablet use, help parents and adolescents navigate through the survey, or answer any general questions about the survey. The parent survey included questions about the following sociodemographic characteristics: age, gender, level of education, household size, income, and time in the US. The adolescent surveys included questions about their age and gender.

The dietary intake of parents and adolescents was assessed with the National Cancer Institute Dietary Screener Questionnaire [41], a short (26-item) instrument used to estimate the frequency of the intake of select food groups (fruit and vegetables, fiber and whole grains, added sugars, dairy, calcium-containing foods, and red meat and processed meat). This questionnaire has been validated for use with the general population and with Hispanics [42,43]. Estimated intake of fruits (cup equivalents), vegetables (including legumes; cup equivalents), dairy (cup equivalents), added sugars (teaspoon equivalents), whole grains (ounce equivalents), fiber (g), and calcium (mg) were calculated using existing algorithms [44]. Questions related to substance use outcomes assessed the use of alcohol in the prior 30 days (“How many times have you consumed alcohol in the past 30 days?”), intentions to use substances (“If you had the chance this weekend, would you use (alcohol, cigarettes, marijuana, inhalants, or e-cigarettes)?”), and personal norms against substance use (“Is it OK for someone your age to (use alcohol, cigarettes, marijuana, inhalants, or e-cigarettes)?”), as previously reported [35,45].

### 2.5. Statistical Analyses

Latent-change modeling, conducted in Mplus 8.4 [46,47,48], was used to assess changes in dietary intake across T1, T2, and T3 for both parents and adolescents and test for the efficacy of the FPNG+ intervention in short-term (T2) and long-term (T3) changes in dietary intake relative to FPNG and RAD. Latent-change models allow for the simultaneous assessment of within-group and between-group changes over time. These models assess the time point at which differences occur, as well as the magnitude and direction of the differences while adjusting for measurement error and reducing estimate bias [48]. Using the Mplus Auxiliary command, full-information maximum likelihood (FIML) [49] estimation was employed to conduct intent-to-treat analyses that adjusted for attrition. Data are presented as means ± SD. The comparative fit index (CFI) was used to evaluate the goodness-of-fit in all models, with a CFI > 0.95 considered to be a good fit [50,51] and >0.90 considered an acceptable fit [52].

Substance use was expected to be low based on prior experience and the age of adolescent participants (12–14 years) [35]. Therefore, answers to each of the questions for individual substances were summed and recoded as a binary yes/no to report any substance use in the past 30 days. Intentions to use and personal norms against substance use were mean scales across all substances. Due to significant sample size limitations related to halting intervention and data collection after the onset of the COVID-19 pandemic, Cohen’s d effect sizes and the 95% confidence interval were calculated for all substance use outcomes, adjusting for missingness using FIML.

## 3. Results

### 3.1. Participant Characteristics

Among participating parents (n = 235; 40.2 ± 6.1 years), the majority were female (89%), originally from Mexico (95%), and married or cohabiting with a partner (87%). More than half of the parent participants had a level of education lower than high school (57%) and reported an annual household income of <USD25,000 (65%). The mean reported household size was 5.3 ± 1.7 people. Among participating adolescents (n = 235; 12.4 ± 0.9 years), more than half were male (60%) and the majority were born in the US (86%). There were no significant differences in participant characteristics among groups at baseline, with the exception of the proportion of participating adolescent boys, which was greater among families enrolled in the FPNG Original program (X2 = 7.63; *p* = 0.02). The sociodemographic characteristics of study participants assigned to each intervention group are listed in Table 1. 

### 3.2. Adolescent Dietary Intake Changes

Table 2 displays the short- and long-term within- and between-group differences in the estimated adolescent intake of vegetables, fruit, whole grains and fiber, total added sugars, added sugars from sugar-sweetened beverages (SSB), and dairy. Adolescents in the FPNG+ group significantly increased their overall intake of fruit, vegetables, and legumes (aggregated) from baseline (T1) to post-intervention (T2, +0.32 cup equivalents, *p* = 0.022). This change was significantly different from the reduction in intake observed among adolescents assigned to RAD (−0.44 cup equivalents, *p* = 0.002 relative to change in FPNG+ group) and FPNG (−0.18 cup equivalents, *p* = 0.006 relative to change in FPNG+ group). This increase was mainly driven by changes in fruit intake, which followed a similar pattern as fruit/vegetables/legumes combined (Table 2). Vegetable/legume intake among adolescents in all groups remained practically unchanged, except for a small reduction among participants in the RAD group at T3 (−0.13 cup equivalents, *p* = 0.024 relative to baseline).

Adolescent whole grains intake was low, estimated as less than an ounce equivalent per day (Table 2). Adolescents in the FPNG+ group decreased their whole grains intake at T3 relative to baseline (−0.16-ounce equivalents, *p* = 0.014), but neither short- nor long-term changes in whole grains intake were significantly different among groups. Whereas adolescents in the RAD group reduced their fiber intake at T2 relative to baseline (−1.78 g, *p* = 0.024), those in the FPNG+ group increased fiber intake at T2 (1.06 g, *p* = 0.048), and these changes from baseline to T2 were significantly different between these two groups (*p* = 0.01). Adolescents in the RAD group further decreased fiber intake at T3 (−2.17 g vs. baseline, *p* = 0.033). Changes in fiber intake from baseline to T3 remained significantly different between RAD and FPNG+ adolescents (*p* = 0.048). Fiber intake remained consistent across all time points for FPNG participants (Table 2).

Estimated total added sugar intake (from all sources) was high for all adolescents throughout the entire study (range: 17.1–18.3 tsp equivalents). Total added sugar intake remained constant over time among adolescents in the FPNG+ group but decreased over time for adolescents in FPNG (−2.36 tsp equivalents at T2 vs. baseline, *p* = 0.003; −2.24 tsp equivalents at T3 vs. baseline, *p* = 0.001) and RAD (−1.56 tsp equivalents at T3 vs. baseline, *p* = 0.001). These reductions over time were significantly different from the lack of change observed among FPNG+ participants (Table 2). There were no significant differences in the changes in the estimated intake of sugar from SSB among adolescents, except for a small decrease in estimated intake among participants in FPNG Original at T3 vs. baseline (−0.59 tsp equivalents, *p* = 0.001).

There were no significant changes in estimated dairy intake over time for adolescents in the FPNG+ group (Table 2). In contrast, estimated dairy intake was significantly lower at T2 and T3 relative to baseline for participants in RAD (−0.25 cup equivalents, *p* = 0.042; −0.24 cup equivalents, *p* = 0.035) and at T3 relative to baseline for FPNG Original participants (−0.46 cup equivalents, *p* = 0.001). Changes at T3 relative to baseline for FPNG Original adolescents were significantly different from the lack of change observed among FPNG+ adolescents (*p* = 0.014).

### 3.3. Parent Dietary Intake Changes

Table 3 displays the short- and long-term within- and between-group differences in the estimated parent intake of vegetables, fruit, whole grains and fiber, total added sugars, added sugars from SSB, and dairy. Like adolescents, parents in the FPNG+ group increased their overall intake of fruit, vegetables, and legumes (aggregated) from baseline to T2 (+0.17 cup equivalents), although this change did not reach statistical significance (*p* = 0.054). In contrast, parents assigned to the other two groups reduced their aggregated fruit/vegetable/legumes intake over time (about −0.2 cup equivalents for both groups at T2 relative to baseline, *p* = 0.001 and 0.014 for RAD and FPNG Original, respectively), and these reductions were significantly different from the trend observed among FPNG+ parents (*p* < 0.005 for both; Table 3). Parents in all groups had lower estimated intakes of aggregated fruit/vegetables/legumes by T3 relative to baseline, but these differences were only statistically significant for parents in the RAD group (*p* = 0.002). Unlike the adolescents, parents in all groups slightly reduced their fruit intake over time. Parents in FPNG+ had a slightly greater, albeit non-statistically significant, estimated intake of vegetables/legumes at T2 (+0.12 cup equivalents vs. baseline) and T3 (+0.05 cup equivalents vs. baseline). These increases in estimated vegetable/legume intake were significantly different from the reductions observed among parents in the RAD group at T2 (−0.09 cup equivalents, n.s. vs. baseline; *p* = 0.048 relative to change in FPNG+ group) and T3 (−0.13 cup equivalents, n.s. vs. baseline; *p* = 0.05 relative to change in FPNG+ group).

Like the adolescents, the parents estimated whole grain intake was less than an ounce equivalent per day. Parents in the FPNG+ group had a greater estimated intake of whole grains at T2 and T3 relative to baseline (+0.25-ounce equivalents at T2 vs. baseline and +0.07-ounce equivalents at T3; *p* < 0.05 for both), whereas whole grain intake decreased over time in RAD participants. Changes in whole grain intake among FPNG+ parents were significantly different from intake changes in RAD parents at T2 and T3 (*p* = 0.001 and *p* = 0.006, respectively) and FPNG Original parents at T2 (*p* = 0.033). There were no significant differences in parent-estimated fiber intake within or among groups (Table 3).

The parent-estimated added sugar intake was slightly lower than that of the adolescents, but still high (range: 14.86–16.67 tsp equivalents). Parents in all groups slightly reduced their added sugar intake over time (Table 3). Relative to changes observed among RAD parents at T2 (−0.68 tsp equivalents vs. baseline), reductions in estimated added sugar intake at T2 were significantly greater among FPNG Original and FPNG+ parents (−3.01 tsp equivalents and −3.22 tsp equivalents; *p* = 0.015 and *p* = 0.021 relative to change in RAD, respectively). T3 estimated added sugar intake was significantly lower than that reported at baseline for all groups (*p* < 0.05 for all), with no differences in changes among parents assigned to different groups. Parents in all groups reduced their estimated intake of sugar from sugar-sweetened beverages over time relative to baseline (*p* < 0.05 for all), with no significant differences in changes among groups (Table 3).

The estimated dairy intake was about 0.1 cup equivalents lower at T2 relative to baseline for all groups (n.s.), and about 0.2 cup equivalents lower at T3 relative to baseline for parents in RAD and FPNG Original (*p* < 0.01 for both). Changes in dairy intake did not significantly differ among parents from different groups (Table 3).

### 3.4. Adolescent-Reported Substance Use Outcomes

Effect sizes of all substance use-related outcomes are reported in Table 4. Relative to adolescents in RAD, both FPNG+ (d = −0.12) and FPNG (d = −0.21) showed small to medium effects for lowered alcohol use at T3 but not at T2. At T2, FPNG+ performed equally well as FPNG (d = 0.0002), but by T3, FPNG showed larger effects for lowered alcohol than FPNG+ (d = 0.17).

Effect sizes for reported intentions to use substances were lower among adolescents in FPNG+ (d = −0.08 and d = −0.06) and FPNG (d = −0.15 and d = −0.12) relative to adolescents in RAD at T2 and T3, respectively; FPNG+ performed equally well to FPNG at T2 and T3 (d = 0.02 and d = 0.01). Effect sizes for personal norms against substance use were stronger for FPNG+ compared to RAD (d = 0.16) and FPNG (d = 0.28); however, by T3, effect sizes for all groups were relatively similar. Due to the trial stoppage because of COVID-19, the small sample size led to confidence intervals that are quite large and contain zero.

## 4. Discussion

The primary objective of the present study was to assess whether it is possible to simultaneously influence healthy eating and substance use prevention through a parenting intervention for Hispanic parents with 6th–8th-grade adolescents. The current study provides evidence that engaging parents in a healthy eating and substance use prevention school-based parenting intervention yielded positive, albeit small, changes in dietary intake among parents and their children and maintained relatively consistent substance use prevention outcomes among adolescents when compared to the original intervention without the nutrition component. Health-related behaviors, including dietary habits, are often established during childhood through parenting practices and behavior management [53]. These behaviors may change over time depending on proximal and distal contextual factors such as the foods that are available to children and adolescents at their homes, parents’ behaviors, and the school environment, among others [24]. Health interventions that offer learning opportunities and the acquisition of new skills and strategies across diverse settings (e.g., home, school) and agents (e.g., parents, teachers) have successfully driven behavior change among children and adolescents [54]; nevertheless, few of these studies were with Hispanic families. One of the key components to achieving positive outcomes in school-based interventions is parental involvement [36,54]. Implementing health-related school-based parenting interventions as a mechanism for behavior change has enabled children and adolescents to maintain or change behaviors in pursuit of positive health outcomes such as healthier eating and delaying substance use [36,53,54].

Nutrition-related FPNG+ content encouraged an increasing intake of fruit, vegetables, and whole grains [40]. Adolescents whose parents were participating in the FPNG+ intervention reported an increase in fruit intake by about 1/3 cup relative to baseline, concomitant with a small increase in fiber intake (about 1 g). NHANES data from the 2017–2018 cycle indicate that mean fruit intake among US adolescents (12–19 years) was about 0.84 cup equivalents [55]. At the public health level, an increase in fruit intake equivalent to the one observed among FPNG+ adolescents would have meaningful implications for population-level adolescent diet quality improvement, particularly when fruit and vegetable intake tends to decline as children transition into adolescence [14]. FPNG+ parents reported small increases in consumption of vegetables and whole grains after the intervention, although overall changes were of a smaller magnitude than those observed among adolescents and not enough to contribute to substantial increases in fiber intake. Nevertheless, it is possible that, albeit small, increases in the vegetable intake of the parents may have been a result of them increasing the availability of fruit and vegetables at home and the parental modeling of consuming these items, which are two of the behaviors encouraged by the FPNG+ intervention that have been reported to have stronger associations with the child’s intake of fruit and vegetables than other parenting feeding practices [25].

The FPNG+ intervention encouraged reducing the intake of added sugars [40]. Adolescents in FPNG+ did not significantly change their added sugar intake after the intervention. Interestingly, adolescents in FPNG and RAD reported a reduction in added sugar intake, which was concomitant with a reduction in dairy intake over time observed for adolescents in all groups. This trend of lower dairy intake as youth age has been previously reported [56], and this is particularly identified for fluid cow’s milk [57]. Whether the reduction in added sugars in FPNG and RAD adolescents was actually due to a reduction in sugar-sweetened dairy products cannot be ascertained using the existing data. Among parents, those in FPNG+ and FPNG reported greater sugar intake reductions post-intervention than parents in RAD, and all parents reported reduced SSB intake over time. The possibility cannot be ruled out that these outcomes may be a function of social desirability derived from repeated dietary assessments over the course of the study.

Whereas the present study was not powered for substance use outcomes due to COVID-19-driven interruptions in study implementation, the preliminary results suggest that adding dietary behavior content to the intervention did not detract from the prevention messages and effects for adolescent substance use. The FPNG+ intervention had comparable effect sizes in reducing and preventing adolescent substance use and lowering intentions to use substances compared to the original FPNG intervention. At T2, the FPNG+ intervention showed larger effect sizes for personal norms against substance use than FPNG.

These findings contribute to theory development by demonstrating that intervening with the family at the microsystem [37,38] can produce multiple desired outcomes simultaneously for the parents and adolescents. As adolescents navigate complex processes such as acculturation at the macrosystem [39], parenting interventions can effectively bridge the norms of the child, the family, and the larger societal norms, producing stronger desired changes in both parents and children, simultaneously, in more than one health outcome.

This research has several limitations worth noting. Diet and substance use data were collected via self-administered surveys, which introduces several sources of bias because of them being self-reported. The current analysis also relied on estimates of intake derived from a short screener developed for the general population that may not have the most representative foods consumed by the target population. Whereas we realize there are important limitations with this methodology, we selected this instrument to reduce participant burden and to ease data collection in a community-based setting, particularly because our targeted sample size was larger than what we were able to attain. However, we had to halt study implementation with the onset of the COVID-19 pandemic, resulting in a smaller sample than originally planned. This in turn limits the power to detect statistical differences and control for baseline differences in adolescent gender in our models as well as make any final statistical conclusions about the efficacy of the intervention related to preventing adolescent substance use. Finally, the FPNG+ parenting intervention has a somewhat limited nutrition scope, which may have contributed to the small magnitude of observed changes.

## 5. Conclusions

In conclusion, our findings suggest that the FPNG+ parenting intervention contributed to some favorable diet changes in the participating adolescents and parents, relative to those in the FPNG Original and comparison intervention groups, while maintaining effects related to substance use outcomes among adolescents. This provides preliminary evidence to support that parenting can be used as a strategy to improve adolescent diet quality and simultaneously prevent the use of substances. Having a parenting intervention that encourages communication, fostering a positive parent–child relationship, and role modeling behaviors to transmit norms for healthy behaviors—both in dietary patterns and anti-substance use norms—can create beneficial changes in adolescents. This study highlights parents’ ability to influence, shape, alter, and change their adolescent’s health behaviors and prevent deleterious health outcomes. Further work is needed to better understand whether parenting-related behaviors contribute to the mechanisms through which the program had favorable effects on both behaviors.

## Figures and Tables

**Table 1:** Participants’ sociodemographic characteristics at baseline by intervention group.

		Parents					Adolescents			
Characteristic	RAD (n = 78)	FPNG (n = 55)	FPNG+ (n = 106)	Statistic	*p* Value	RAD (n = 78)	FPNG (n = 55)	FPNG+ (n = 106)	Statistic	*p* Value
Age (y)	40.0 ± 6.6	41.7 ± 6.3	39.7 ± 5.5	F = 1.99	*p =* 0.14	12.2 ± 0.9	12.5 ± 1.1	12.4 ± 0.9	F = 1.34	*p =* 0.26
Household size	5.3 ± 2.0	5.2 ± 1.3	5.3 ± 1.7	F = 0.24	*p =* 0.79					
Gender				χ^2^ = 2.17	*p =* 0.34				χ^2^ = 7.63	*p* = 0.02
Male	7.7%	9.1%	14.2%			60.3%	45.5%	67.9%		
Female	92.3%	90.9%	85.8%			39.7%	54.5%	32.1%		
Country of birth				χ^2^ = 4.23	*p =* 0.38				χ^2^ = 6.90	*p* = 0.14
United States	3.8%	3.6%	4.8%			82.1%	92.7%	85.9%		
Mexico	82.1%	89.1%	89.5%			12.8%	7.3%	13.2%		
Other	14.1%	7.3%	5.7%			5.1%	0.0%	0.9%		
Marital Status				χ^2^ = 0.95	*p =* 0.62					
Married/cohabitating	85.7%	90.7%	85.6%							
Single/divorced	14.3%	9.3%	14.4%							
Education				χ^2^ = 2.65	*p* = 0.62					
Less than high school	64.0%	56.6%	52.4%							
High school or GED	18.7%	22.6%	27.2%							
More than high school	17.3%	20.8%	20.4%							
Annual household income				χ^2^ = 16.18	*p* = 0.18					
Less than USD10,000	21.8%	14.8%	13.7%							
USD10,000–USD14,999	20.5%	9.3%	16.7%							
USD15,000–USD19,999	15.4%	14.8%	8.8%							
USD20,000–USD24,999	19.2%	24.0%	18.6%							
USD25,000–USD29,999	6.4%	14.8%	7.8%							
USD30,000–USD49,999	12.8%	13.0%	24.5%							
USD50,000 or higher	3.9%	9.3%	9.8%							

**Table 2:** Short- and long-term within- and between-group differences in the estimated intake of select food groups and nutrients derived from the Dietary Screening Questionnaire among participating adolescents ^a^.

Food Group/Nutrient	Mean ± SE			Short-Term		Long-Term	
	T1	T2	T3	T2-T1	*p*	T3-T1	*p*
Fruit/Vegetables/Legumes (cup equivalents)							
RAD	2.46 ± 0.36	2.02 ± 0.18	1.82 ± 0.12	−0.44	0.034	−0.64	0.015
FPNG	2.09 ± 0.28	1.91 ± 0.17	1.86 ± 0.04	−0.18	0.116	−0.23	0.332
FPNG+	2.22 ± 0.09	2.54 ± 0.22	1.97 ± 0.08	0.32	0.022	−0.25	0.048
FPNG vs. RAD				0.26	0.277	0.42	0.252
FPNG+ vs. RAD				0.76	0.002	0.40	0.179
FPNG+ vs. FPNG				0.50	0.006	−0.02	0.943
Vegetables plus Legumes (cup equivalents)							
RAD	1.11 ± 0.10	1.06 ± 0.12	0.98 ± 0.06	−0.05	0.299	−0.13	0.024
FPNG	1.14 ± 0.18	1.10 ± 0.08	0.92 ± 0.05	−0.05	0.686	−0.22	0.110
FPNG+	1.28 ± 0.10	1.27 ± 0.11	1.10 ± 0.04	−0.02	0.720	−0.19	0.112
FPNG vs. RAD				0.00	0.987	−0.09	0.562
FPNG+ vs. RAD				0.03	0.611	−0.06	0.667
FPNG+ vs. FPNG				0.03	0.810	0.03	0.852
Fruit (cup equivalents)							
RAD	1.32 ± 0.19	1.07 ± 0.13	0.81 ± 0.06	−0.24	0.021	−0.51	0.001
FPNG	1.16 ± 0.15	0.94 ± 0.14	0.91 ± 0.01	−0.22	0.001	−0.25	0.113
FPNG+	0.99 ± 0.04	1.31 ± 0.18	0.92 ± 0.07	0.32	0.026	−0.07	0.361
FPNG vs. RAD				0.02	0.817	0.26	0.230
FPNG+ vs. RAD				0.56	0.002	0.44	0.007
FPNG+ vs. FPNG				0.54	0.001	0.18	0.299
Whole grains (ounce equivalents)							
RAD	0.62 ± 0.12	0.63 ± 0.08	0.53 ± 0.04	0.01	0.822	−0.08	0.549
FPNG	0.53 ± 0.01	0.48 ± 0.04	0.49 ± 0.04	−0.06	0.197	−0.04	0.119
FPNG+	0.76 ± 0.09	0.79 ± 0.13	0.60 ± 0.04	0.03	0.532	−0.16	0.014
FPNG vs. RAD				−0.07	0.303	0.04	0.773
FPNG+ vs. RAD				0.02	0.801	−0.08	0.616
FPNG+ vs. FPNG				0.09	0.168	−0.12	0.099
Fiber (g)							
RAD	16.02 ± 1.15	14.24 ± 0.81	13.85 ± 0.33	−1.78	0.024	−2.17	0.033
FPNG	13.93 ± 0.74	13.69 ± 0.53	13.66 ± 0.14	−0.23	0.640	−0.27	0.666
FPNG+	14.88 ± 0.25	15.94 ± 0.67	14.96 ± 0.38	1.06	0.048	0.08	0.863
FPNG vs. RAD				1.55	0.120	1.90	0.085
FPNG+ vs. RAD				2.84	0.010	2.25	0.048
FPNG+ vs. FPNG				1.30	0.056	0.35	0.638
Added sugars (tsp equivalents)							
RAD	17.97 ± 0.41	17.98 ± 1.27	16.41 ± 0.42	0.01	0.997	−1.56	0.001
FPNG	18.51 ± 0.51	16.15 ± 0.48	16.27 ± 0.14	−2.36	0.003	−2.24	0.001
FPNG+	18.00 ± 0.73	18.42 ± 1.10	18.14 ± 0.71	0.42	0.613	0.14	0.846
FPNG vs. RAD				−2.36	0.116	−0.68	0.308
FPNG+ vs. RAD				0.41	0.799	1.69	0.021
FPNG+ vs. FPNG				2.77	0.028	2.37	0.010
Added sugars from SSB (tsp equivalents)							
RAD	8.44 ± 0.74	7.82 ± 0.49	7.47 ± 0.31	−0.62	0.305	−0.97	0.126
FPNG	7.98 ± 0.16	7.61 ± 0.17	7.39 ± 0.08	−0.37	0.246	−0.59	0.001
FPNG+	8.10 ± 0.24	8.96 ± 0.601	8.31 ± 0.57	0.86	0.185	0.21	0.673
FPNG vs. RAD				0.26	0.709	0.38	0.558
FPNG+ vs. RAD				1.48	0.109	1.17	0.142
FPNG+ vs. FPNG				1.22	0.067	0.79	0.123
Dairy(cup quivalents)							
RAD	1.95 ± 0.10	1.71 ± 0.11	1.71 ± 0.05	−0.25	0.042	−0.24	0.035
FPNG	2.09 ± 0.13	2.05 ± 0.04	1.63 ± 0.07	−0.05	0.859	−0.46	0.001
FPNG+	1.87 ± 0.15	1.73 ± 0.09	1.90 ± 0.07	−0.13	0.415	0.06	0.740
FPNG vs. RAD				0.20	0.495	−0.22	0.210
FPNG+ vs. RAD				0.12	0.561	0.30	0.139
FPNG+ vs. FPNG				−0.09	0.788	0.52	0.014

^a^ All models have a CFI > 0.95.

**Table 3:** Short- and long-term within- and between-group differences in estimated intake of select food groups and nutrients derived from the Dietary Screening Questionnaire among participating parents ^a^.

Food Group/Nutrient	Mean ± SE			Short-Term		Long-Term	
	T1	T2	T3	T2-T1	*p*	T3-T1	*p*
Fruit/Vegetables/Legumes (cup equivalents)							
RAD	2.46 ± 0.36	2.02 ± 0.18	1.82 ± 0.12	−0.44	0.034	−0.64	0.015
FPNG	2.09 ± 0.28	1.91 ± 0.17	1.86 ± 0.04	−0.18	0.116	−0.23	0.332
FPNG+	2.22 ± 0.09	2.54 ± 0.22	1.97 ± 0.08	0.32	0.022	−0.25	0.048
FPNG vs. RAD				0.26	0.277	0.42	0.252
FPNG+ vs. RAD				0.76	0.002	0.40	0.179
FPNG+ vs. FPNG				0.50	0.006	−0.02	0.943
Vegetables plus Legumes (cup equivalents)							
RAD	1.11 ± 0.10	1.06 ± 0.12	0.98 ± 0.06	−0.05	0.299	−0.13	0.024
FPNG	1.14 ± 0.18	1.10 ± 0.08	0.92 ± 0.05	−0.05	0.686	−0.22	0.110
FPNG+	1.28 ± 0.10	1.27 ± 0.11	1.10 ± 0.04	−0.02	0.720	−0.19	0.112
FPNG vs. RAD				0.00	0.987	−0.09	0.562
FPNG+ vs. RAD				0.03	0.611	−0.06	0.667
FPNG+ vs. FPNG				0.03	0.810	0.03	0.852
Fruit (cup equivalents)							
RAD	1.32 ± 0.19	1.07 ± 0.13	0.81 ± 0.06	−0.24	0.021	−0.51	0.001
FPNG	1.16 ± 0.15	0.94 ± 0.14	0.91 ± 0.01	−0.22	0.001	−0.25	0.113
FPNG+	0.99 ± 0.04	1.31 ± 0.18	0.92 ± 0.07	0.32	0.026	−0.07	0.361
FPNG vs. RAD				0.02	0.817	0.26	0.230
FPNG+ vs. RAD				0.56	0.002	0.44	0.007
FPNG+ vs. FPNG				0.54	0.001	0.18	0.299
Whole grains (ounce equivalents)							
RAD	0.62 ± 0.12	0.63 ± 0.08	0.53 ± 0.04	0.01	0.822	−0.08	0.549
FPNG	0.53 ± 0.01	0.48 ± 0.04	0.49 ± 0.04	−0.06	0.197	−0.04	0.119
FPNG+	0.76 ± 0.09	0.79 ± 0.13	0.60 ± 0.04	0.03	0.532	−0.16	0.014
FPNG vs. RAD				−0.07	0.303	0.04	0.773
FPNG+ vs. RAD				0.02	0.801	−0.08	0.616
FPNG+ vs. FPNG				0.09	0.168	−0.12	0.099
Fiber (g)							
RAD	16.02 ± 1.15	14.24 ± 0.81	13.85 ± 0.33	−1.78	0.024	−2.17	0.033
FPNG	13.93 ± 0.74	13.69 ± 0.53	13.66 ± 0.14	−0.23	0.640	−0.27	0.666
FPNG+	14.88 ± 0.25	15.94 ± 0.67	14.96 ± 0.38	1.06	0.048	0.08	0.863
FPNG vs. RAD				1.55	0.120	1.90	0.085
FPNG+ vs. RAD				2.84	0.010	2.25	0.048
FPNG+ vs. FPNG				1.30	0.056	0.35	0.638
Added sugars (tsp equivalents)							
RAD	17.97 ± 0.41	17.98 ± 1.27	16.41 ± 0.42	0.01	0.997	−1.56	0.001
FPNG	18.51 ± 0.51	16.15 ± 0.48	16.27 ± 0.14	−2.36	0.003	−2.24	0.001
FPNG+	18.00 ± 0.73	18.42 ± 1.10	18.14 ± 0.71	0.42	0.613	0.14	0.846
FPNG vs. RAD				−2.36	0.116	−0.68	0.308
FPNG+ vs. RAD				0.41	0.799	1.69	0.021
FPNG+ vs. FPNG				2.77	0.028	2.37	0.010
Added sugars from SSB (tsp equivalents)							
RAD	8.44 ± 0.74	7.82 ± 0.49	7.47 ± 0.31	−0.62	0.305	−0.97	0.126
FPNG	7.98 ± 0.16	7.61 ± 0.17	7.39 ± 0.08	−0.37	0.246	−0.59	0.001
FPNG+	8.10 ± 0.24	8.96 ± 0.601	8.31 ± 0.57	0.86	0.185	0.21	0.673
FPNG vs. RAD				0.26	0.709	0.38	0.558
FPNG+ vs. RAD				1.48	0.109	1.17	0.142
FPNG+ vs. FPNG				1.22	0.067	0.79	0.123
Dairy (cup equivalents)							
RAD	1.95 ± 0.10	1.71 ± 0.11	1.71 ± 0.05	−0.25	0.042	−0.24	0.035
FPNG	2.09 ± 0.13	2.05 ± 0.04	1.63 ± 0.07	−0.05	0.859	−0.46	0.001
FPNG+	1.87 ± 0.15	1.73 ± 0.09	1.90 ± 0.07	−0.13	0.415	0.06	0.740
FPNG vs. RAD				0.20	0.495	−0.22	0.210
FPNG+ vs. RAD				0.12	0.561	0.30	0.139
FPNG+ vs. FPNG				−0.09	0.788	0.52	0.014

^a^ All models have a CFI > 0.95.

**Table 4:** Effect sizes of adolescent-reported alcohol use in the past 30 days, intentions to use substances, and personal norms against substance use across intervention conditions.

Variable	FPNG+ vs. RAD		FPNG vs. RAD		FPNG+ vs. FPNG	
	d	95% C.I.	d	95% C.I.	d	95% C.I.
Alcohol use in the past 30 days						
Effect at T2	0.05	(−0.24, 0.35)	0.04	(−0.31, 0.38)	0.0002	(−0.33, 0.33)
Effect at T3	−0.12	(−0.46, 0.12)	−0.21	(−0.59, 0.10)	0.17	(−0.11, 0.54)
Intentions to use any substance						
Effect at T2	−0.08	(−0.37, 0.21)	−0.15	(−0.49, 0.20)	0.02	(−0.31, 0.34)
Effect at T3	−0.06	(−0.35, 0.23)	−0.12	(−0.47, 0.22)	0.01	(−0.31, 0.34)
Personal substance use norms						
Effect at T2	0.16	(−0.14, 0.45)	−0.11	(−0.46, 0.23)	0.28	(−0.05, 0.61)
Effect at T3	0.05	(−0.25, 0.34)	0.06	(−0.29, 0.40)	−0.01	(−0.34, 0.31)

## Data Availability

The data presented in this study are available on request from the corresponding author. The data will be made available upon request pending application, approval, and establishment of a corresponding data-sharing agreement.

## References

[1] U.S. Census Bureau (2021). Supplementary Tables on Race and Hispanic Origin: 2020 Census Redistricting Data.

[2] Caballero A.E. (2005). Diabetes in the Hispanic or Latino population: Genes, environment, culture, and more. Curr. Diab Rep..

[3] Zhang H., Rodriguez-Monguio R. (2012). Racial disparities in the risk of developing obesity-related diseases: A cross-sectional study. Ethn. Dis..

[4] Leventhal A.M., Huh J., Dunton G.F. (2014). Clustering of modifiable biobehavioral risk factors for chronic disease in us adults: A latent class analysis. Perspect. Public. Heal..

[5] Pi-Sunyer X. (2009). The medical risks of obesity. Postgrad. Med..

[6] Ogden C.L., Carroll M.D., Fakhouri T.H., Hales C.M., Fryar C.D., Li X., Freedman D.S. (2018). Prevalence of obesity among youths by household income and education level of head of household—United States 2011–2014. MMWR Morb. Mortal Wkly Rep..

[7] Power T.G., Hidalgo-Mendez J., Fisher J.O., O’Connor T.M., Micheli N., Hughes S.O. (2020). Obesity risk in Hispanic children: Bidirectional associations between child eating behavior and child weight status over time. Eat. Behav..

[8] Fryar C.D., Carroll M.D., Afful J. (2020). Prevalence of overweight, obesity, and severe obesity among children and adolescents aged 2–19 years: United States, 1963–1965 through 2017–2018. *NCHS Health E-Stats*. https://www.cdc.gov/nchs/data/hestat/obesity-child-17-18/obesity-child.htm.

[9] Fanelli S.M., Jonnalagadda S.S., Pisegna J.L., Kelly O.J., Krok-Schoen J.L., Taylor C.A. (2020). Poorer diet quality observed among us adults with a greater number of clinical chronic disease risk factors. J. Prim. Care Community Health.

[10] Guillermo C., Boushey C.J., Franke A.A., Monroe K.R., Lim U., Wilkens L.R., Le Marchand L., Maskarinec G. (2020). Diet quality and biomarker profiles related to chronic disease prevention: The multiethnic cohort study. J. Am. Coll. Nutr..

[11] Krebs-Smith S.M., Pannucci T.E., Subar A.F., Kirkpatrick S.I., Lerman J.L., Tooze J.A., Wilson M.M., Reedy J. (2018). Update of the healthy eating index: HEI-2015. J. Acad. Nutr. Diet..

[12] Krebs-Smith S.M., Guenther P.M., Subar A.F., Kirkpatrick S.I., Dodd K.W. (2010). Americans do not meet federal dietary recommendations. J. Nutr..

[13] Overcash F., Reicks M. (2021). Diet quality and eating practices among Hispanic/Latino men and women: NHANES 2011–2016. Int. J. Environ. Res. Public Health.

[14] U.S. Department of Agriculture, Food and Nutrition Service, Promotion, C.f.N.P.A (2021). Average Healthy Eating Index-2015 Scores for Americans by Race/Ethnicity, Ages 2 Years and Older. What We Eat in America, NHANES 2017–2018. https://www.fns.usda.gov/cnpp/hei-scores-americans.

[15] Xu F., Cohen S.A., Greaney M.L., Hatfield D.L., Greene G.W. (2019). Racial/ethnic disparities in us adolescents’ dietary quality and its modification by weight-related factors and physical activity. Int. J. Environ. Res. Public. Health.

[16] Steinberger J., Daniels S.R., Hagberg N., Isasi C.R., Kelly A.S., Lloyd-Jones D., Pate R.R., Pratt C., Shay C.M., Towbin J.A. (2016). Cardiovascular health promotion in children: Challenges and opportunities for 2020 and beyond. A Scientific Statement From the American Heart Association. Circulation.

[17] Rosinger A., Herrick K., Gahche J., Park S. (2017). Sugar-sweetened beverage consumption among U.S. Adults, 2011–2014. NCHS Data Brief.

[18] Bekelman T.A., Johnson S.L., Taylor C.A. (2020). Differences in diet quality and snack intakes among non-Hispanic white and Mexican American adolescents from different acculturation groups. J. Racial Ethn. Health Disparities.

[19] Wang L., Martinez Steele E., Du M., Pomeranz J.L., O’Connor L.E., Herrick K.A., Luo H., Zhang X., Mozaffarian D., Zhang F.F. (2021). Trends in consumption of ultraprocessed foods among us youths aged 2–19 years, 1999–2018. JAMA.

[20] Oviedo-Solis C.I., Monterrubio-Flores E.A., Cediel G., Denova-Gutierrez E., Barquera S. (2022). Trend of ultraprocessed product intake is associated with the double burden of malnutrition in Mexican children and adolescents. Nutrients.

[21] Shamah-Levy T., Cuevas-Nasu L., Méndez-Gómez-Humarán I., Jimenez-Aguilar A., Mendoza-Ramirez A.J., Villalpando S. (2011). [Obesity in Mexican school age children is associated with out-of-home food consumption: In the journey from home to school]. Arch. Latinoam. Nutr..

[22] Wang Y., Beydoun M.A., Li J., Liu Y., Moreno L.A. (2011). Do children and their parents eat a similar diet? Resemblance in child and parental dietary intake: Systematic review and meta-analysis. JECH.

[23] Robinson L., Rollo M., Watson J., Burrows T., Collins C. (2015). Relationships between dietary intakes of children and their parents: A cross-sectional, secondary analysis of families participating in the family diet quality study. J. Hum. Nutr. Diet..

[24] Scaglioni S., De Cosmi V., Ciappolino V., Parazzini F., Brambilla P., Agostoni C. (2018). Factors influencing children’s eating behaviours. Nutrients.

[25] Yee A.Z., Lwin M.O., Ho S.S. (2017). The influence of parental practices on child promotive and preventive food consumption behaviors: A systematic review and meta-analysis. IJBNPA.

[26] Davison K.K., Lawson H.A., Coatsworth J.D. (2012). The family-centered action model of intervention layout and implementation (famili): The example of childhood obesity. Health Promot. Pract..

[27] Klohe-Lehman D.M., Freeland-Graves J., Clarke K.K., Cai G., Voruganti V.S., Milani T.J., Nuss H.J., Proffitt J.M., Bohman T.M. (2007). Low-income, overweight and obese mothers as agents of change to improve food choices, fat habits, and physical activity in their 1-to-3-year-old children. J. Am. Coll. Nutr..

[28] Harrell J.S., Gansky S.A., McMurray R.G., Bangdiwala S.I., Frauman A.C., Bradley C.B. (1998). School-based interventions improve heart health in children with multiple cardiovascular disease risk factors. Pediatrics.

[29] Slawta J., Bentley J., Smith J., Kelly J., Syman-Degler L. (2008). Promoting healthy lifestyles in children: A pilot program of be a fit kid. Health Promot. Pr. Pract..

[30] Arcan C., Neumark-Sztainer D., Hannan P., van den Berg P., Story M., Larson N. (2007). Parental eating behaviours, home food environment and adolescent intakes of fruits, vegetables and dairy foods: Longitudinal findings from project eat. Public Health Nutr..

[31] Macauly A.P., Griffin K.W., Gronewold E., Williams C., Botvin G.J. (2005). Parenting practices and adolescent drug-related knowledge, attitudes, norms, and behavior. J. Alcohol. Drug Educ..

[32] Ramirez J.R., Crano W.D., Quist R., Burgoon M., Alvaro E.M., Grandpre J. (2004). Acculturation, familism, parental monitoring, and knowledge as predictors of marijuana and inhalant use in adolescents. Psychol. Addict. Behav..

[33] Marsiglia F.F., Williams L.R., Ayers S.L., Booth J.M. (2014). Familias: Preparando la Nueva Generacion: A randomized control trial testing the effects on positive parenting practices. Res. Soc. Work. Pract..

[34] Williams L.R., Ayers S.L., Garvey M.M., Marsiglia F.F., Castro F.G. (2012). Efficacy of a culturally based parenting intervention: Strengthening open communication between Mexican-heritage parents and adolescent children. J. Soc. Soc. Social. Work. Res..

[35] Marsiglia F.F., Ayers S.L., Baldwin-White A., Booth J. (2016). Changing Latino adolescents’ substance use norms and behaviors: The effects of synchronized youth and parent drug use prevention interventions. Prev. Sci..

[36] Williams L.R., Ayers S., Baldwin A., Marsiglia F.F. (2016). Delaying youth substance-use initiation: A cluster randomized controlled trial of complementary youth and parenting interventions. J. Soc. Soc. Work. Res..

[37] Bronfenbrenner U. (1986). Ecology of the family as a context for human development: Research perspectives. Dev. Psychol..

[38] Bronfenbrenner U. (1979). The Ecology of Human Development: Experiments by Nature and Design.

[39] Prado G., Huang S., Maldonado-Molina M., Bandiera F., Schwartz S.J., de la Vega P., Brown C.H., Pantin H. (2010). An empirical test of ecodevelopmental theory in predicting hiv risk behaviors among Hispanic youth. Health Educ. Behav..

[40] Vega-López S., Marsiglia F.F., Ayers S., Williams L.R., Bruening M., Gonzalvez A., Vega-Luna B., Perilla A., Harthun M., Shaibi G.Q. (2020). Methods and rationale to assess the efficacy of a parenting intervention targeting diet improvement and substance use prevention among latinx adolescents. Contemp. Clin. Trials.

[41] Thompson F.E., Midthune D., Subar A.F., McNeel T., Berrigan D., Kipnis V. (2005). Dietary intake estimates in the national health interview survey, 2000: Methodology, results, and interpretation. J. Am. Diet. Assoc..

[42] Colón-Ramos U., Thompson F.E., Yaroch A.L., Moser R.P., McNeel T.S., Dodd K.W., Atienza A.A., Sugerman S.B., Nebeling L. (2009). Differences in fruit and vegetable intake among Hispanic subgroups in California: Results from the 2005 California Health Interview Survey. J. Am. Diet. Assoc..

[43] Yaroch A.L., Tooze J., Thompson F.E., Blanck H.M., Thompson O.M., Colon-Ramos U., Shaikh A.R., McNutt S., Nebeling L.C. (2012). Evaluation of three short dietary instruments to assess fruit and vegetable intake: The National Cancer Institute’s Food Attitudes and Behaviors Survey. J. Acad. Nutr. Diet..

[44] Thompson F.E., Midthune D., Kahle L., Dodd K.W. (2017). Development and evaluation of the National Cancer Institute’s Dietary Screener Questionnaire scoring algorithms. J. Nutr..

[45] Kulis S., Marsiglia F.F., Elek E., Dustman P., Wagstaff D.A., Hecht M.L. (2005). Mexican/Mexican American adolescents and Keepin’ it Real: An evidence-based substance use prevention program. Child. Sch..

[46] Muthén L.K., Muthén B.O. (2015). Mplus: Statistical Analysis with Latent Variables.

[47] Muthén L.K., Muthén B.O. (2017). Mplus User’s Guide.

[48] McArdle J.J. (2009). Latent variable modeling of differences and changes with longitudinal data. Annu. Rev. Psychol..

[49] Graham J.W. (2009). Missing data analysis: Making it work in the real world. Annu. Rev. Psychol..

[50] Hu L., Bentler P. (1999). Cutoff criteria for fit indexes in covariance structure analysis: Conventional criteria versus new alternatives. Struct. Equ. Model..

[51] West S.G., Taylor A.B., Wu W., Hoyle R. (2012). Model fit and model selection in structural equation modeling. Handbook of Structural Equation Modeling.

[52] McDonald R.P., Ho M.H. (2002). Principles and practice in reporting structural equation analyses. Psychol. Methods.

[53] Montano Z., Smith J.D., Dishion T.J., Shaw D.S., Wilson M.N. (2015). Longitudinal relations between observed parenting behaviors and dietary quality of meals from ages 2 to 5. Appetite.

[54] Dogan R.K., King M.L., Fischetti A.T., Lake C.M., Mathews T.L., Warzak W.J. (2017). Parent-implemented behavioral skills training of social skills. J. Appl. Behav. Anal..

[55] Bowman S.A., Clemens J.C., Friday J.E. (2021). Food pattern group and macronutrient intakes of adolescents 12 to 19 years: WWEIA, NHANES 2003–2004 to 2017–2018. FSRG Dietary Data Briefs.

[56] Hohoff E., Perrar I., Jancovic N., Alexy U. (2021). Age and time trends of dairy intake among children and adolescents of the DONALD study. Eur. J. Nutr..

[57] Stewart H., Kuchler F., Dong D., Cessna J. (2021). Examining the Decline in U.S. per Capita Consumption of Fluid Cow’s Milk, 2003–2018, ERR-300.

